# To Dr. Bradley

**Published:** 1805-07-01

**Authors:** James Hamilton

**Affiliations:** Artillery Place


### To Dr. BRADLEY.

Sir,

THE following mode of preparing a saline draught will be found to produce one as agreeable, and in some cases more efficacious than the one in general use, composed of the carbonate of potass and the citric acid. The citric acid is often scarce; sometimes impure and expensive: the ingredients of the other are always to be had; they are cheap, and never vary in quality.

℞. Sub-boras sodæ gr. xv. super-tartris potassæ ʒß. solve terendo in mortareo cum lact. amygd. ʒx. dein adde syr. simpl. aq. cinnam. a. ʒj. f. haust.

If you should think the above prescription worthy of being more generally known, have the goodness to insert it in the Medical and Physical Journal.

Yours, &c.

*Artillery Place, June* 17, 1105. JAMES HAMILTON.

